# First-Trimester Ultrasound: A Comparative Review of Guidelines

**DOI:** 10.3390/diagnostics16111695

**Published:** 2026-05-30

**Authors:** Eirini Boureka, Ioannis Tsakiridis, Georgios Michos, Anastasios Liberis, Sonia Giouleka, Apostolos Mamopoulos, Ioannis Kalogiannidis, Themistoklis Dagklis

**Affiliations:** Third Department of Obstetrics and Gynecology, School of Medicine, Faculty of Health Sciences, Aristotle University of Thessaloniki, Konstantinoupoleos 49, 54642 Thessaloniki, Greece; ebourek@auth.gr (E.B.); gmichos@auth.gr (G.M.); alymperi@auth.gr (A.L.); soniagiouleka@outlook.com (S.G.); amamop@auth.gr (A.M.); ikalogia@auth.gr (I.K.); dagklis@auth.gr (T.D.)

**Keywords:** first-trimester ultrasound, nuchal translucency, screening, fetal anatomy, guidelines, comparison

## Abstract

First-trimester sonographic examination remains a fundamental part of antenatal care, providing crucial information for the well-being of both the mother and fetus and leading to the best possible perinatal outcomes. This study aimed to review and compare the most recently published guidelines on first-trimester ultrasound. Therefore, a descriptive review of guidelines from the American Institute of Ultrasound in Medicine (AIUM), the Australasian Society of Ultrasound in Medicine (ASUM), the Association of the Scientific Medical Societies in Germany (AWMF), the International Society of Ultrasound in Obstetrics and Gynecology (ISUOG), the Society of Obstetrician and Gynecologists of Canada (SOGC) and the World Association of Perinatal Medicine (WAPM) regarding first-trimester scans was conducted. There is a consensus regarding the main parameters that should be evaluated, the qualifications of the examiner and specifications of the ultrasound machine, as well as the importance of informed consent. Additionally, the importance of careful visualization of fetal anatomy is discussed, with minor discrepancies regarding the appropriate protocol used. The use of combined first-trimester examination is encouraged by all the reviewed medical societies, although cell-free DNA testing is addressed only by a few, with similar indications. Controversy exists regarding the indications and appropriate gestational age at which the first-trimester scan should be performed, as well as the proper establishment of pregnancy dating. Notably, recommendations regarding fetal growth restriction (FGR) and diagnostic invasive procedures are made only by few medical societies, with the AWMF and SOGC addressing screening for FGR. Furthermore, invasive diagnostic testing is discussed by the AIUM, AWMF and SOGC, with differentiations among them regarding the indications for pursuing such procedures. First-trimester sonographic examination is essential for assessing fetal viability, establishing accurate pregnancy dating, evaluating fetal and maternal anatomy and calculating the risk of various fetal and maternal conditions. The implementation of evidence-based, unified protocols would advance both maternal and fetal outcomes.

## 1. Introduction

The first-trimester scan is a fundamental part of antenatal care, focused on the evaluation of early pregnancy [1]. It is typically performed between 11^+0^ and 13^+6^ weeks and serves as a valuable tool for the confirmation of intrauterine pregnancy; evaluation of the presence, location and number of gestational sacs; determination of gestational age; assessment of fetal number, chorionicity and amnionicity; and evaluation of early fetal anatomy [2].

Both transvaginal and transabdominal approaches can be utilized, with transvaginal ultrasound offering superior resolution for early gestational structures when transabdominal imaging is inconclusive [3]. Systematic evaluation of fetal anatomy during this period allows for the identification of major structural anomalies, although until recently basic fetal anatomical assessments were primarily performed during the second trimester, which served as the gold standard [4].

The first-trimester scans has also contributed significantly to the timely assessment of chromosomal abnormalities, allowing for early decision-making and intervention opportunities [5]. Measurement of the nuchal translucency (NT), along with maternal serum biomarkers or cell-free DNA (cfDNA) testing, provides a sensitive screening approach for common aneuploidies. Increased NT is the most frequent abnormal finding in the first trimester and may prompt further genetic evaluation with invasive testing [6].

These facts underline the importance of developing consistent international evidence-based algorithms for proper sonographic evaluation during the first trimester of pregnancy to provide clear guidance to healthcare professionals and ensure optimal maternal and fetal care. Hence, the aim of this descriptive review was to synthesize and compare the recommendations from influential guidelines concerning the performance of first-trimester ultrasound examination.

## 2. Evidence Acquisition

The most recently published guidelines on the first-trimester ultrasound examination were retrieved and a comparative review was conducted. More specifically, six guidelines were identified from: the American Institute of Ultrasound in Medicine (AIUM 2021) [7], the Australasian Society of Ultrasound in Medicine (ASUM 2018) [8], the Association of the Scientific Medical Societies in Germany (AWMF 2025) [9,10], the International Society of Ultrasound in Obstetrics and Gynecology (ISUOG 2023) [11], the Society of Obstetrician and Gynecologists of Canada (SOGC 2021) [12] and the World Association of Perinatal Medicine (WAPM 2022) [13].

An overview of the recommendations is presented in Table 1 (general ultrasound principles) and Table 2 (combined first-trimester screening and specific examinations).

## 3. General Principles

### 3.1. Scan

The first-trimester scan is used to evaluate several parameters of an ongoing pregnancy: the fetal viability (AIUM, ASUM, ISUOG, and SOGC), fetal number (AIUM, ASUM, ISUOG, and SOGC), pregnancy dating (AIUM, ASUM, AWMF, ISUOG, SOGC, and WAPM), fetal anatomy (AIUM, ASUM, AWMF, ISUOG, SOGC, and WAPM), location of the gestational sac (ASUM, ISUOG) and, in case of multiple pregnancy, evaluation of the amnionicity and chorionicity (AIUM, ASUM, ISUOG, and SOGC).

The AWMF, ISUOG, SOGC and WAPM agree regarding the indications for performing this examination. In detail, these medical societies consider the first-trimester ultrasound as a fundamental part of routine antenatal care, recommending it for all pregnant women [1]. In contrast, the AIUM suggests that it should be reserved only for groups at high risk for skeletal anomalies, while the ASUM mentions that it can be performed upon request. Furthermore, the WAPM guidelines specifically mention that a detailed fetal anatomical assessment protocol should be undertaken only in high-risk cases, although a brief anatomical evaluation is recommended in all pregnancies. This recommendation is based on a consideration of factors, such as examination time, maternal anxiety and cost-effectiveness when conducting an ultrasound scan. Published data showed that the detection rate of structural anomalies in high-risk women was 61% (95% CI: 37.71–82.19%) compared to only 32% in low-risk pregnancies (95% CI: 22.45–43.12%) [14]. Of note, the WAPM guidelines focus mainly on fetal anatomy protocols, and therefore limited recommendations on other first-trimester parameters are provided.

With regards to twin pregnancies, results from a population study showed that the detection rate of anatomic anomalies was only 27.3% (95% CI: 15.0–42.8), highlighting the need for improved prenatal screening protocols and specialized ultrasound surveillance in multiple gestations [15].

The reviewed medical societies display some discrepancies regarding the recommended timing for performing the first-trimester ultrasound scan. Most guidelines suggest conducting it between 11^+0^ and 13^+6^ gestational weeks (AWMF, ISUOG, SOGC, and WAPM). This is because all the above-mentioned characteristics can be examined optimally on a larger fetus in the late first trimester [2,16]. However, other guidelines suggest narrower or broader timeframes; the AIUM recommends a stricter window from 12^+0^ to 13^+6^ weeks, while the ASUM mentions a much wider range, allowing for the examination to be performed anytime between 5 and 14 weeks of gestation. With regards to the AIUM, given the fact that this medical society recommends a detailed sonographic scan to be performed only in high-risk pregnancies, the limited timeframe is justified, as with a larger fetus the structural anomalies become easier to identify. On the other hand, the ASUM refers to the first-trimester ultrasound evaluation in general and accepts its performance throughout the whole trimester. Moreover, it mentions specific instructions for practitioners as to which parameters require evaluation depending on each gestational week.

There is agreement among the reviewed guidelines with regards to the prerequisites needed before conducting this specific examination. All medical societies state that it should be performed by specialized staff with expertise in fetal ultrasound and particular training from reputable organizations [17]. Moreover, both transabdominal and transvaginal transducers need to be available, as results from a systematic review showed that the combination of transabdominal and transvaginal examination resulted in a significantly higher detection rate (62% compared to 51%—abdominal and 34%—transvaginal, *p* < 0.001, respectively) [18]. Especially regarding obese populations, a recent study showed that a transvaginal approach was required in 63% of cases to complete the anatomy views during the first-trimester scan [19]. Additionally, emphasis is placed on obtaining appropriate patient consent (AIUM, ASUM, and AWMF) and recording the patient history (ASUM) prior to the examination.

### 3.2. Pregnancy Dating

Several discrepancies were identified in relation to the assessment of pregnancy dating. More specifically, the AIUM and ASUM base their antenatal care on the date of the last menstrual period (LMP), while the AWMF, ISUOG and SOGC consider the crown–rump length (CRL) to be the most accurate method for determining gestational age in spontaneously conceived pregnancies [20]. This is based on a study that showed that the prediction error of CRL between 8 and 16 weeks was 7.7 days, while the error by LMP was a little more than 10 days; therefore, the CRL is considered a more accurate method [21]. However, both the AIUM and ASUM accept the CRL as an alternative method when discrepancies exist between ultrasound measurements and the LMP. In detail, the AIUM recommends using the CRL if a difference of more than 7 days from the LMP is identified between 9 and 13^+6^ gestational weeks, whereas the ASUM advises its use in cases of unknown LMP, a difference of more than 5 days prior to 10 gestational weeks, or more than 7 days between 10 and 14 weeks of gestation. In the latter case, the ASUM specifies that the biparietal diameter (BPD) can be used to assess gestational age, complementary to the CRL. According to results from a study of 3655 women, determination of gestational age by the LMP resulted in more post-term deliveries compared to the CRL (when it differed from the LMP by more than 7 days) [22]. Moreover, when the algorithm using the CRL measurement, with a difference of more than 7 days from the LMP, was followed, the number of deliveries within one week from the estimated date was higher compared to that using the LMP (57.1% vs. 48.1%) [22].

Of note, the CRL is a reliable method only when it measures between 45 and 84 mm. This is justified by a prospective study that showed that with advancing gestation the inter-observer variability in the CRL increased from +1.42 to −2.15 at 55 mm to +3.12 to −2.36 at 80 mm [23]. Consequently, the SOGC suggests using alternative biometric indices for fetuses with a CRL greater than 84 mm.

Notably, there is a consensus among the reviewed guidelines, except for the WAPM, which does not address the topic, regarding the determination of gestational age in pregnancies conceived via assisted reproductive technology (ART). In these cases, the gestational age and estimated due date (EDD) are calculated based on data obtained from the in vitro fertilization (IVF) procedures. This is based on data from a retrospective study that showed that the CRL in IVF pregnancies resulted in a mean overestimation of gestational age by 3 days (95% CI: 2.7–3.4, *p* < 0.001) [24].

As for multiple pregnancies resulting from spontaneous conception, the AIUM, ASUM and SOGC recommend using the CRL of the larger fetus to determine the gestational age. This approach is intended to avoid overlooking early fetal growth restriction (FGR).

### 3.3. Fetal Biometry

In addition to the measurement of CRL for pregnancy dating, the reviewed guidelines suggest evaluation of fetal biometry with the measurement of several parameters. Firstly, the CRL is proposed by all guidelines, except the WAPM, as previously mentioned. Notably, the CRL measurement, following a standardized technique, is recommended by all medical societies [25,26]. In detail, the suggested method to correctly measure the CRL is to place the long axis of the fetus perpendicular to the ultrasound beam, in midsagittal position and at full magnification, with the profile, spine and rump visible (AIUM, ISUOG, and SOGC). Moreover, the calipers should be placed on the end points of the crown and rump, with the fetus in neutral position, and the mean of three acceptable measurements (AIUM) or the best measurement (ISUOG) is reported.

Moreover, measurement of BPD, head circumference (HC), abdominal circumference (AC) and femoral length (FL) are considered components of a full biometry evaluation. More specifically, the BPD alone (ASUM, AWMF) or in combination with the HC (AIUM, ISUOG, SOGC, and WAPM) are considered the parameters for evaluating the fetal head. Special consideration should be given to the measurement technique of BPD, as it can be measured either by placing the calipers on the outer-to-inner edge of the calvarial bone or outer-to-outer in accordance with the nomogram employed [27,28]. This is performed on the axial plane of the fetal head, with the ultrasound beam perpendicular to the midline falx and on full magnification. Additionally, the thalami should be visualized, along with the third ventricle (AIUM, ISUOG, and WAPM).

Additionally, the AC is a part of routine fetal biometry according to the AIUM, ISUOG, SOGC, and WAPM, either as an eclipse or with the measurement of the antero-posterior abdominal diameter (APAD) and transverse abdominal diameter (TAD), as proposed alternatively by the ISUOG, while the AWMF mentions that it can be measured optionally. More specifically, the fetal abdomen should be placed on the axial scan, in full magnification, with the stomach and, ideally, the intrahepatic portion of the portal vein visible. Additionally, one vertebral body should be seen at 3 or 9 o’clock, and a single rib on each side of the fetal abdomen. Of note, studies have shown that measurement of the AC demonstrates high intra- and inter-observer precision and should be considered an integral component of first-trimester fetal evaluation [29,30].

Finally, the FL is recommended by the AIUM, ISUOG and SOGC as a routine measurement, while the WAPM and AWMF consider it optional. Both recommendations are justified, as the FL shows high variability in its measurement and therefore cannot be used reliably for gestational age [27]. However, it remains a useful tool for the assessment of fetal skeletal dysplasias [31]. With regards to the optimal plane for FL measurement, it should be fully magnified to occupy the majority of the image; the ultrasound beam should be placed perpendicular to its long axis and the calipers placed on each end of the ossified diaphysis.

### 3.4. Fetal Anatomy

One of the most important parts of the first-trimester sonographic examination is the evaluation of the fetal anatomy, a subject discussed by all the reviewed medical societies. This is performed based on standard planes to assess each anatomic region, as shown in Table 3. In fact, results from recent studies showed that with proper clinical training, the first-trimester ultrasound scan proves both clinically and cost-effective for diagnosing major fetal structural anomalies [32,33], especially when using specific standardized protocols [34]. Most guidelines agree that the fetal anatomy evaluation should include examination of the head and brain (AIUM, ASUM, AWMF, and WAPM); the face and profile (AIUM, AWMF), or at least the ossification of the jaw (ASUM); the neck, heart, abdomen and extremities (AIUM, ASUM, AWMF, ISUOG, WAPM); the urogenital tract (AWMF, WAPM), the spine (AIUM, ASUM, AWMF, and WAMP); and thorax (AIUM, AWMF, and WAPM). Of note, the ISUOG mentions that the minimum requirements of the fetal anatomy evaluation should include the head and brain, neck, heart, abdomen and extremities, while a more detailed scan should include the face, thorax, genitalia and spine. The SOGC, on the other hand, does not specifically describe the anatomical structures that should be examined. Instead, this medical society outlines the conditions that are always or often detectable and should therefore be actively assessed. These are classified as always detectable, including acrania, holoprosencephaly, encephalocele, pentalogy of Cantrell, ectopia cordis, exomphalos, gastroschisis and body stalk anomaly; and often detectable, including spina bifida, complex heart defect, lower urinary tract obstruction, absence of extremities and fatal skeletal dysplasia. Consequently, the SOGC indirectly implies that the head and brain, neck, heart, abdomen, urogenital tract, spine and extremities should be evaluated during the first-trimester scan.

During the examination of the fetal head, the axial, sagittal and transventricular planes should be assessed. Moreover, the transthalamic plane, as well as the angling of the axial plane towards the posterior fossa, are important images for a careful evaluation of the fetal brain. Careful assessment of the choroid plexus morphology should be undertaken, as the “butterfly sign” has a sensitivity and specificity of 100% (95% CI: 71.3–100% and 99.9–100%, respectively) for detecting holoprosencephaly [35]. This is also highlighted by a study that showed that when using a standardized protocol, the detection of central nervous system anomalies presented with a specificity of 72.7% and a sensitivity of 100% [36], with several anatomic protocols proposed accordingly [37]. Moreover, according to a large study, careful visualization of the skull, midline echo, choroid plexuses, midbrain and brain stem resulted in identification of all cases of acrania, holoprosencephaly, and encephalocele, as well as 59% of open spina bifida cases and 13% of cerebellum abnormalities [2]. Additionally, abnormalities in the intracranial translucency and the fetal posterior fossa anatomy are associated with a Dandy–Walker malformation or other cerebral abnormalities [38]. Similarly, results from a retrospective study demonstrated that in open spina bifida cases, the crash sign (i.e., the posterior displacement of the mesencephalon and deformation against the occipital bone) was present in 90.6% of cases, showing a strong correlation with this specific medical condition [39].

Moreover, the face and profile should be evaluated by visualization of the forehead, nose, maxilla, mandible and mouth. This is performed with the fetus in midsagittal view, with maximum magnification of the fetal profile; with the axial frontal view, for assessment of the fetal orbits; and with the coronal plane for evaluation of the bony palate. Studies have shown that assessment of the maxillary gap for the “superimposed-line” sign are useful markers for identification of cleft lip and/or palate [40,41]. More specifically, a maxillary gap was seen in 96% of cases with cleft lip and/or palate with additional anomalies, and in 65% the finding was isolated [40]. Furthermore, the “superimposed-line” sign showed a sensitivity and specificity of 89.5% and 99.8%, respectively, for detecting orofacial clefts according to a prospective study of almost 10,000 fetuses [41]. This was also highlighted by a recent meta-analysis, which showed that the overall detection rate of cleft palates was as high as 0.874 during the first-trimester ultrasound scan [42]. Additionally, the mandibular gap was present in 77.8% of cases with micrognathia, posing as a possible ultrasound marker for this structural anomaly [43]. Subsequently, the nasal bone should be evaluated on the midsagittal view of the fetal head, as its inclusion in first-trimester protocols for major chromosomal defects screening led to an overall detection rate of 92% with a false-positive rate of 2.9% [44]. Interestingly, the AIUM, AWMF and ISUOG suggest routine evaluation of the above-mentioned sonographic markers, with the AIUM and ISUOG including assessment of the orbits, lenses and upper lip. In contrast, the WAPM mentions only evaluation of the fetal profile as part of the routine examination, while assessment of the eye orbits, bony palate and upper lip are not routinely recommended.

Continuing with the fetal neck, careful evaluation should be performed to identify the presence of cystic hygroma, dilated jugular lymphatic sacs or any other discrete fluid-filled collection or masses. In addition, a thorough measurement of the nuchal translucency (NT) should be obtained, as suggested by all the reviewed medical societies. This is performed with the fetus on a midsagittal plane, with its neck in a neutral position, perpendicular to the ultrasound beam. According to a large study of almost 10,000 women, the NT was above the 95th centile in 82.2% of fetuses with trisomy 21 and 77.9% with other chromosomal defects, therefore presenting as a valuable marker for identification of fetal abnormalities [45].

The fetal heart and thorax are important components of the first-trimester anatomical evaluation. The fetal lungs, cardiac position, situs, size and cardiac axis should be carefully observed, as well as the four-chamber view of the fetal heart. The cardiac axis is an important parameter for cardiac defects, as results from a multicenter study showed that it was abnormal in 74.1% of fetuses with congenital heart disease [46]. Moreover, application of color Doppler significantly improved visualization of the fetal heart in early pregnancy compared with 2D imaging, especially relating to the inferior vena cava, pulmonary veins and aortic and ductal arches [47]. Additionally, visualization of the three-vessel and trachea should be attempted, when technically feasible, although it is not mandatory, as discussed by the AIUM, AWMF and WAPM. Of note, color Doppler contributes significantly to the visualization of both outflow tracts before 10 gestational weeks, as detection increased from only 16% with 2D imaging to 64% with color Doppler [47]. This was also highlighted by a recent meta-analysis, which showed that first-trimester early fetal echocardiography achieved a pooled sensitivity and specificity of 0.838 and 1.000, respectively, for diagnosing major fetal heart defects [48]. The optimal plane to assess the above-mentioned anatomic areas is the axial fetal plane, with one rib present on each side of the fetal thorax, and preferably with an apical view of the fetal heart so as to assess the ventricular septum correctly. Continuing from the four-chamber apical view, the sonographer should sweep the probe cranially to evaluate the arches and complete the heart examination. Moreover, a transverse view with the ultrasound beam parallel to the ventricular and atrial septum is also considered an acceptable alternative.

Furthermore, the abdominal wall should be examined, especially in relation to the umbilical cord insertion, the stomach location and any potential abdominal cysts. This is performed with the fetus in axial or sagittal views, with or without the addition of color Doppler to support the examination. Data from a cohort study showed that while the majority of cysts resolved antenatally, with no remaining consequences for the neonate, four cases with lower abdomen cysts were diagnosed with anorectal malformations postnatally [49]. Additionally, mid-gut herniation is associated with gestational age, presenting in approximately 60% of fetuses before 10 weeks and in only 5% at 12 weeks, a fact that should be considered when evaluating the abdominal wall [50]. Notably, the WAPM mentions that evaluation of the bowel is not recommended as routine during this ultrasound scan. Nevertheless, if any abnormal or suspicious findings are observed, a further advanced diagnostic assessment should be undertaken. Specifically, hyperechogenic fetal bowel is associated with several fetal pathological conditions, such as cystic fibrosis, chromosomal anomalies, Zellweger syndrome, etc., all of which require advanced management at a specialized center [51].

Along the same route, the pelvis requires careful observation, especially in relation to the kidneys, the bladder and the genitalia. As described by the AIUM, both kidneys should be evaluated. If difficulty arises in identifying them, the use of color Doppler on the axial plane to assess the renal arteries is recommended in cases of inadequate renal visualization. Results from a large study demonstrated that only a small number of cases with renal anomalies were detected in the first trimester [2]. However, as some conditions can be fatal for a fetus, such as bilateral renal agenesis, careful examination is optimal. On the other hand, lower urinary tract obstruction presents with a much higher detection rate (71.2%) during the first-trimester anatomic scan [2]. Moreover, results from a prospective study involving nearly 60,000 women showed that the prevalence of megacystis was 1 in 1632, with a chromosomal abnormality detected in 31.4% of affected cases [52]. Furthermore, the bladder diameter plays a crucial role in determining the fetal outcome: a longitudinal bladder diameter of 7 to 15 mm was associated with an abnormal karyotype in approximately one out of every four cases, but spontaneous resolution occurred in nearly 90% of these cases [53]. In contrast, when the diameter exceeded 15 mm, all examined cases were associated with obstructive uropathy [53]. The fetal bladder can be examined either on axial or sagittal planes, but in cases of enlargement the measurement should be performed on the midsagittal view only. Notably, visualization of the genitalia is not mandatory; however, it can be evaluated when feasible on a midsagittal view.

As previously mentioned, the fetal limbs should be routinely assessed for abnormalities during the first-trimester anatomic survey. This is justified by the results of a study where almost 65% of cases with skeletal abnormalities were identified during the first trimester [54]. In detail, the presence of all three segments of upper and lower limbs should be confirmed and any potential rotation or bending should be excluded. Notably, the WAPM specifically mentions that measurement of the femur and humerus is not recommended during this examination. However, data from a study showed that the BPD/FL ratio can aid in the diagnosis of severe skeletal dysplasias, such as thanatophoric dysplasia [31]. Therefore, the AIUM, ISUOG and SOGC support the inclusion of this measurement as routine. Notably, a detailed evaluation of the fingers and toes is not recommended and should be restricted to only in high-risk cases for specific musculoskeletal disorders.

Specific recommendations regarding the anatomic evaluation of the fetal spine are made by the AIUM, AWMF, ISUOG and WAPM. In detail, the vertebral alignment and the skin integrity should be examined, ideally in the midsagittal view. Although the coronal view is considered easier to obtain, it is less useful for the evaluation of the fetal skin. Notably, the spine should be carefully evaluated in cases where spina bifida is suspected from the presence of anomalies in the posterior fossa [55,56].

### 3.5. Placenta and Umbilical Cord

The placenta is considered as an important structure to access during the first-trimester ultrasound. All the reviewed guidelines referring to the topic mention that its texture, location and insertion should be examined. Notably, ISUOG recommends a complete assessment of the placenta during the detailed first-trimester scan, while the minimum requirements consider evaluation of the placental texture alone as sufficient. Results from a study showed that in cases of triploidy, the placenta presented with molar changes in approximately 16% of cases [57,58]. Additionally, among pregnancies affected by mesenchymal placental dysplasia, only 9% of them resulted in a good outcome [59]. Therefore, the importance of placental texture is highlighted, and should be included on a routine scan. Furthermore, the placental location in relation to the cervical os should be observed. However, cases of placenta previa should not be noted as a definitive diagnosis, as it was that among 42.3% cases of placenta previa at 11 to 14 gestational weeks, only 1.9% were indeed diagnosed at term [60]. Moreover, in cases with a previous cesarean delivery, careful evaluation of the placenta to exclude scar pregnancy and placenta accreta spectrum disorders is crucial [61]. Results from a meta-analysis showed that sonographic signs of abnormally invasive placenta can be present from the first trimester, with the gestational sac close to the previous cesarean scar present in 82.4% (95% CI: 46.6–99.8%) of cases, lacunae in 46% (95% CI: 10.9–83.7%) and reduced myometrial thickness in 66.8% (95% CI: 45.2–85.2%) of cases [62]. Furthermore, low implantation of the gestational sac presented with an almost 20-times higher risk of abnormally invasive placenta (OR: 19.6; 95% CI: 6.7–57.3) [62].

As highlighted by the AIUM, AWMF and ISUOG, the umbilical cord insertion should be looked for in the first-trimester sonographic assessment. Data from a prospective study showed that low cord insertion presented in approximately 12% of pregnancies, out of which 4.7% presented with velamentous cord insertion compared to the normal ones (OR: 6.67; 95% CI: 2.67–16.63) [63]. Additionally, pregnancies with low cord insertion presented with a significantly higher risk of vasa previa (*p* = 0.001) compared to central umbilical insertion [64]. Notably, velamentous cord insertion is significantly associated with adverse pregnancy outcomes, such as preeclampsia, stillbirth, placental abruption and small-for-gestational-age neonates, as shown by a meta-analysis [65].

### 3.6. Uterus and Adnexa

Regarding the evaluation of the maternal structures, the AIUM, ASUM and ISUOG recommend routine assessment of the uterus, mainly for the presence of fibroids and congenital malformations. Furthermore, measurement of the cervical length is recommended routinely by the AIUM, while the AWMF, ISUOG and SOGC suggest its optional evaluation. According to a large-scale study, short cervical length in the first trimester combined with maternal characteristics was significantly associated with preterm delivery [66]. Notably, the ASUM mentions that cervical evaluation should be conducted after 14 gestational weeks.

Moreover, visualization of the ovaries and adnexa is described by the AIUM, ASUM and SOGC, with the AIUM additionally recommending evaluation of the cul-de-sac. Ovarian masses are a relatively common finding in pregnancy, although they tend to resolve spontaneously during the antenatal period [67,68].

## 4. Additional Examinations

### 4.1. Maternal Biochemistry

Evaluation of maternal biochemistry in combination with ultrasound parameters serves as a helpful tool for calculating the risk for several chromosomal anomalies; routine maternal free beta-human chorionic gonadotropin (β-hCG) and pregnancy-associated plasma protein A (PAPP-A) are recommended by the ASUM, AWMF, ISUOG and SOGC, while the ISUOG additionally mentions the utility of placental growth factor (PlGF). According to a large prospective study of almost 20,000 women, a combination of maternal age, fetal NT, maternal free β-hCG and PAPP-A resulted in detection rate of trisomy 21 of 90% (95% CI: 84–96%) with a false-positive rate of 3%, compared to only 81% (95% CI: 73–89%) for screenings with maternal age and fetal NT and 63% (95% CI: 56–72%) for screenings with maternal age and maternal serum biochemistry alone [69]. Notably, the PlGF concentration was significantly associated with fetuses with trisomy 21 (*p* < 0.0001), but its use is targeted mainly for the detection of preeclampsia in high-risk patients [70]. More specifically, a combination of uterine arteries’ Doppler, mean arterial pressure (MAP) and PlGF resulted in a prediction of 90% for early, 75% for preterm and 41% for term preeclampsia [71].

### 4.2. Nuchal Translucency

The NT is an important parameter of the first-trimester ultrasound examination, serving as an ultrasound marker for many chromosomal and anatomic anomalies. All medical societies referring to the topic (ASUM, AWMF, ISUOG, and SOGC) suggest that its evaluation should be performed according to the Fetal Medicine Foundation’s (FMF’s) protocol [72]. Moreover, the ASUM, ISUOG, SOGC and WAPM emphasize the importance of having trained personnel conduct this specific examination. More specifically, the CRL of the examined fetus should be between 45 mm and 84 mm according to the protocol described by Nicolaides et al. [45]. In detail, the fetus should be examined in midsagittal plane, in a neutral position with a magnification that includes only the fetal head and thorax. The NT is measured at its maximum thickness with calipers measuring 0.1 mm increments. Of note, this protocol, in combination with maternal biochemistry, identifies approximately 90% of fetuses with trisomy 21, with a false positive rate of 5% [72]. On the other hand, the NT in combination with maternal age has a sensitivity of 82.2% and a negative predictive value of 99.9% [45].

### 4.3. Additional Ultrasound Markers

Apart from the NT measurement, several other parameters serve as additional ultrasound markers, contributing to the algorithm for the diagnosis of chromosomal anomalies. Firstly, the flow through the tricuspid valve is suggested by the AWMF, ISUOG, WAPM and SOGC (only in high-risk women). More specifically, the tricuspid flow is assessed by visualizing the four-chamber view of the heart, while placing it on the 6 or 12 o’clock position. Moreover, a 2–4 mm pulse wave is placed on the tricuspid valve to produce tricuspid flow. Results from a study showed that tricuspid regurgitation was significantly associated with trisomy 21, 18, 13 and Turner syndrome [73]. Moreover, when the examination of tricuspid valve flow was added to the algorithm (maternal age, NT, fetal heart rate, serum β-hCG and PAPP-A), the detection rate for trisomy 21 was 96%, while the detection rates for trisomy 18, 13 and Turner syndrome were 92%, 100% and 100%, respectively [73].

With regards to the ductus venosus flow, it is recommended as a routine by the AWMF and ISUOG and for high-risk women by the SOGC, whereas the WAPM states that this assessment should not be performed routinely. The ductus venosus is examined with the fetus in a right paramedial section and support from color Doppler. According to a study, a reversed a-wave on the ductus venosus was observed in 66.4% of fetuses with trisomy 21 and 75% of those with Turner syndrome [74]. Additionally, co-evaluation of this parameter along with maternal age, NT, maternal free h-CG, PAPP-A and fetal heart rate increases the detection rates of trisomy 21, 18, 13 and Turner syndrome to 96%, 92%, 100% and 100%, respectively [74].

Furthermore, the examination of nasal bone is routinely recommended by the ISUOG and in intermediate-risk women by the AWMF, while the SOGC does not recommend its routine evaluation. Its evaluation is performed on the same midsagittal view as the NT, with the tip of the nose and the rectangular shape of the palate visible. Its examination is justified based on a large-scale study which showed that absent or hypoplastic nasal bone was associated with trisomy 21 in almost 60% of cases, while the detection rate increased to 91% when combined with the above-mentioned screening protocol [44].

Notably, the WAPM recommends routine evaluation of the cranial posterior fossa, as published data have shown that abnormalities in this anatomic area are associated with open spina bifida in the majority of cases and therefore serves as a valuable ultrasound marker of spinal abnormalities [75].

### 4.4. Screening for Preeclampsia and Fetal Growth Restriction

Specific recommendations regarding screening for preeclampsia and fetal growth restriction (FGR) during the first-trimester scan are provided by the AWMF, ISUOG and SOGC. As previously mentioned, during the first-trimester sonographic examination, the screening method that combines maternal characteristics, MAP, and maternal biochemistry (free β-hCG, PAPP-A and PlGF) is performed. More specifically, the combination of the above-mentioned, with the exception of PAPP-A, leads to the prediction of early, preterm and term preeclampsia in Caucasian women at rates of 88%, 69% and 40%, respectively, while for women of Afro-Caribbean origin, the rates are 100%, 92% and 75%, respectively [71]. Of note, the AWMF, ISUOG and SOGC mention that screening for preeclampsia should be made according to the FMF protocol, with minor discrepancies among them. More specifically, the AWMF and ISUOG suggest the combination of maternal history, uterine artery Pulsatility Index (UtAPI), and maternal biochemistry, with the ISUOG specifically mentioning only PlGF and MAP. Furthermore, the ISUOG specifically mentions screening in limited settings, with a combination of maternal history, maternal characteristics and MAP, without performing maternal biochemistry examinations, or reserving them for specific women at high-risk only. This is based on a study that showed that a two-step screening protocol led to a small decrease in detection rate of preterm preeclampsia, from 74% to 71% [76]. Similarly, the SOGC suggests routine screening for the prediction of preeclampsia, but mentions that UtA-PI should not be routinely included in the algorithm. Of note, the AWMF and ISUOG mention that in high-risk women, with a risk of more than 1 in 100 of developing preeclampsia, the administration of 150 mg of aspirin daily should be recommended until 36 gestational weeks [77]. This is based on the ASPRE study, which showed that in women at high-risk of preeclampsia who received low-dose aspirin from 11 to 14 gestational weeks until 36 weeks, the risk of developing preeclampsia was significantly decreased (OR: 0.38; 95% CI: 0.20–0.74; *p* = 0.004) [78].

Moreover, with regards to small-for-gestational-age (SGA) fetuses and prediction of fetal growth restriction (FGR), the SOGC mentions that a combination of maternal characteristics, history, MAP and UtA-PI, although not as a routine, should be used, while the ISUOG mentions that Doppler examinations can be helpful in their prediction. Data have shown that when using a combination of parameters, the detection rate of preterm-SGA and term-SGA was 55.5% and 44.3%, respectively [79].

### 4.5. Cell-Free DNA Testing

Continuing with a more advanced screening during the first trimester, cfDNA testing is mentioned only by the AWMF and ISUOG. In detail, both medical societies agree that it should be used as a screening method for common aneuploidies, while the AWMF is against its use for rare, structural anomalies or for microdeletions, duplications or monogenic defects. On the other hand, the ISUOG mentions that cfDNA examination may be extended to cover these conditions in the future. This recommendation is based on a large study which showed that, when intermediate-risk women were screened with cfDNA, the detection rate of trisomy 21 was 98.4% with a false-positive rate of 0.7% [80]. This was also highlighted by a meta-analysis that demonstrated that in singletons, screening with cfDNA resulted in the detection of fetuses with trisomy 21, 18 and 13 at even higher rates of more than 99%, 98% and 99%, respectively, with a false-positive rate of 0.13% [81]. Moreover, for the detection of rarer chromosomal anomalies, the positive predictive value was estimated at 32% for structural aberrations, highlighting the need for a careful assessment of the benefit–risk ratio before recommending universal implementation of cfDNA screening for all chromosomal abnormalities [82]. Notably, the AWMF mentions that the test should be repeated in 2 weeks in case of inconclusive initial results, based on a study that showed that in a failed cfDNA test, a repeated test provided a result in 62.8% of cases [83].

### 4.6. Invasive Diagnostic Procedures

Invasive testing is discussed by the AIUM, AWMF and SOGC, with different recommendations among them. The AIUM mentions that it should be suggested in cases where fetal anomalies are detected in early gestation, the SOGC recommends it in fetuses with an NT ≥ 3.5 mm, whereas the AWMF recommends it in cases with fetal malformations, an NT ≥ 3.0 mm and especially if ≥3.5 mm, abnormal maternal biochemistry or abnormal cfDNA results [84]. This is based on a study that showed that for an NT over 3.5 mm, pathogenic variants were detected in 13.8% of fetuses, as well as in 6.5% of cases with an NT over 3.0 mm [85]. Moreover, in cases with PAPP-A < 0.2 MoM, the prevalence of atypical abnormal karyotype was 4.17 (95% CI: 3.07–5.65), while for free β-hCG < 0.2 MoM the relevant prevalence was 7-times higher (95% CI: 4.39–11.14) [86]. Similarly, according to a study, in 3.7% of women undergoing cfDNA the results were inconclusive, and therefore confirmation of the findings with invasive testing was necessary [87].

## 5. Upgrade of Guidelines

Among the reviewed guidelines, several have been updated from previous versions throughout the years. More specifically, the SOGC guidelines are updated from the previous version published in 2001 and provide information regarding the evaluation for the risk of preeclampsia and SGA, the assessment of a multiple pregnancy and other pregnancy complications, and guidelines on the examination of additional ultrasound markers (tricuspid and ductus venosus flow and nasal bone). Similarly, the AWMF guidelines, which are an upgrade of 2018 version, provide more detailed information regarding overall fetal and maternal risk stratification (aneuploidies, preeclampsia, SGA, etc.) and pregnancy management, whereas the 2018 guidelines focused mainly on aneuploidy screening and cfDNA implementation. Finally, the ISUOG guidelines are also upgraded since 2013, introducing both a minimum-required and a detailed fetal anatomic protocol, as well as a systematic evaluation of intracranial anatomy, additional ultrasound markers, placental assessment and uterine artery Doppler, while also presenting the importance of screening for obstetric complications, such as preterm preeclampsia. All the above-mentioned updated versions focus on a more comprehensive fetal and maternal assessment, supporting earlier detection of fetal anomalies and allowing for more individualized antenatal management and follow-up.

## 6. Conclusions

In summary, all the reviewed guidelines agree on the main parameters that should be examined, including determination of fetal number and viability, accurate pregnancy dating, detailed evaluation of the fetal anatomy and assessment of chorionicity and amnionicity in multiple gestations. Moreover, agreement exists regarding the experience and qualifications of the examiner, the use of both transvaginal and transabdominal transducers when required, and the necessity of obtaining informed patient consent. Furthermore, there is an overall consensus regarding the evaluation of the fetal anatomy, with most guidelines recommending examination of similar anatomical areas. An exception exists regarding the ISUOG and WAPM guidelines, which propose a minimum-requirements protocol that includes only a limited set of anatomical areas, as well as a more detailed scan protocol. Notably, the SOGC does not specify the exact anatomical structures to be assessed, but instead classifies structural defects based on how frequently they are detected during the first-trimester scan. Similarly, with regards to the placenta, uterus and adnexa, there is a general agreement among the reviewed medical societies, with some requiring a more detailed examination, and others a more basic one. Homophony exists regarding the use of combined first-trimester screening with maternal biochemistry; NT measurement; and additional ultrasound markers, such as the nasal bone, ductus venosus and tricuspid flow, with the medical societies that address the topic highlighting its clinical importance not only for identification of chromosomal defects but also for calculating the risk of preeclampsia. Finally, cfDNA is addressed only by the AWMF and ISUOG, with similar recommendations regarding its appropriate use and associated cautions.

On the other hand, discrepancies exist among the reviewed guidelines regarding the indications and appropriate gestational age at which the first-trimester scan should be performed. Moreover, differentiation exists regarding the proper management of gestational dating, with the AIUM and ASUM mentioning pregnancy dating based on the LMP as a first choice, whereas the AWMF, ISUOG and SOGC consider the CRL measurement more accurate in cases of spontaneous conception. Similarly, regarding fetal biometry, the AC, HC and FL are not considered necessary parameters by all guidelines. Notably, recommendations regarding FGR and diagnostic invasive procedures are made only by a few medical societies. More specifically, FGR is addressed by the AWMF and SOGC, with the SOGC providing a more comprehensive protocol for screening for FGR during the first trimester. Furthermore, invasive diagnostic testing is discussed by the AIUM, AWMF and SOGC, with differences among them regarding the indications for performing such procedures.

In our opinion, the ISUOG 2023 guidelines represent one of the most comprehensive and standardized approaches to first-trimester ultrasound screening. Compared with the above-mentioned guidelines, they place greater emphasis on detailed early fetal anatomic assessment, integration of Doppler and placental evaluation, and standardized imaging protocols. Furthermore, their broad international use has supported their implementation across healthcare systems with different organizational structures, levels of expertise and available resources.

In conclusion, first-trimester ultrasound examination is essential for assessing fetal viability, establishing accurate pregnancy dating, evaluating fetal and maternal anatomy and calculating the risk of various fetal and maternal conditions. Therefore, the implementation of evidence-based, unified protocols is essential for improving pregnancy outcomes, reducing perinatal mortality and providing early options for couples. Thus, well-structured screening programs would significantly reduce the burden of fetal disease, and play a pivotal role in improving both maternal and fetal outcomes.

## Figures and Tables

**Table 1:** General ultrasound principles.

	AIUM	ASUM	AWMF	ISUOG	SOGC	WAPM
**Country**	United States	Australia	Germany	International	Canada	International
**Issued**	2020	April 2021	2024	2023	August 2021	2022
**Title**	AIUM Practice Parameter for the Performance of Detailed Diagnostic Obstetric Ultrasound Examinations Between 12 Weeks 0 Days and 13 Weeks 6 Days	Guidelines for the Performance of First-Trimester Ultrasound	First-Trimester Diagnosis and Therapy @ 11–13^+6^ Weeks of Gestation—Part 1 First-Trimester Diagnosis and Therapy @ 11–13^+6^ Weeks of Gestation—Part 2	ISUOG Practice Guidelines: Performance of 11–14 Week Ultrasound Scan	Committee Opinion No. 418: The Complete 11–14 Week Prenatal Sonographic Examination	First-Trimester Examination of Fetal Anatomy: Clinical Practice Guidelines by the World Association of Perinatal Medicine (WAPM) and the Perinatal Medicine Foundation (PMF)
**Pages**	16	10	14/17	17	9	15
**References**	124	9	401	109	50	68
**Examination**	Pregnancy dating Fetal number Fetal viability Fetal anatomy In multiple gestation, amnionicity/chorionicity	Pregnancy dating Gestational sac Fetal number Fetal viability Fetal anatomy In multiple gestation, amnionicity/chorionicity	Pregnancy dating Fetal anatomy Fetal NT Fetal/maternal Doppler	Pregnancy dating Gestational sac Fetal number Fetal viability Fetal anatomy In multiple gestation, amnionicity/chorionicity	Pregnancy dating Fetal number Fetal viability Fetal anatomy In multiple gestation, amnionicity/chorionicity	Pregnancy dating Fetal anatomy
**Indications**	High-risk groups	Upon request	All pregnant women	All pregnant women	All pregnant women	All pregnant women Detailed protocol for high-risk groups
**Gestational age**	12^+0^ to 13^+6^ wks	5 to 14 wks	11^+0^ to 13^+6^ wks	11^+0^ to 14^+0^ weeks	11^+0^ to 14^+0^ weeks	11^+0^ to 13^+6^ weeks
**Prerequisites**	Specialized staff Transvaginal + transabdominal transducer Patient consent	Specialized staff Transvaginal + transabdominal transducer Patient consent Patient history	Specialized staff Transvaginal + transabdominal transducer Patient consent	Specialized staff and transvaginal + transabdominal transducer	Specialized staff and transvaginal + transabdominal transducer	Specialized staff and transvaginal + transabdominal transducer
**Pregnancy dating**	1. LMP. 2. CRL. → if difference > 7 days (from 9 to 13 + 6 wks). 3. IVF data from ART. In multiple gestation, based on CRL of larger fetus.	1. LMP. 2. CRL. → if unknown LMP. → difference > 5 days <10 wks. → (+/− BPD) difference > 7 days between 10 and 14 wks. 3. IVF data from ART. In multiple gestation, based on CRL of larger fetus.	1. CRL. 2. IVF data from ART.	1. CRL (45–84 mm). 2. IVF data from ART.	1. CRL (45–84 mm). 2. IVF data from ART. 3. If CRL > 84 mm, use οther biometric indices. In multiple gestation, based on CRL of larger fetus.	Not mentioned
**Fetal biometry**	CRL BPD and/or HC AC FL	CRL BPD	CRL BPD Optional: HC, AC, FL	CRL BPD and HC AC (or APAD/TAD) FL	CRL BPD/HC AC FL	BPD/HC AC FL not routinely recommended
**Fetal anatomy**	Head and brain Face and profile Neck Heart and thorax Abdomen and pelvis Spine and extremities	Head and brain Jaw Heart Abdomen and pelvis Spine and extremities	Head and brain Face and profile Neck Heart and thorax Abdomen Urogenital tract Spine and extremities	**Minimum requirements:** Head and brain Neck Heart Abdomen Extremities **Detailed scan:** Head and brain Face and neck Thorax and heart Abdomen Genitalia Spine and extremities	**Always detectable:** Acrania, holoprosencephaly, encephalocele, pentalogy of Cantrell, ectopia cordis, exomphalos, gastroschisis and body stalk anomaly **Often detectable:** Spina bifida, complex heart defect, lower urinary tract obstruction, absence of extremities and fatal skeletal dysplasia	Head and brain Face Neck Heart and thorax GIT and abdominal wall Urinogenital tract Spine and extremities
**Placenta**	Texture Location Cord insertion	Not mentioned	Structure Location Cord insertion Amniotic fluid If low umbilical cord insertion, → transvaginal U/S with color Doppler	Minimum requirements: Texture Detailed scan: Size/texture Location Cord insertion Amniotic fluid	Texture Location in previous cesarian	Not mentioned
**Uterus and adnexa**	Uterus Cervix Ovaries Adnexa Cul-de-sac	Uterus Cervix (>14 wks) Ovaries Adnexa	Optional Cervix	Uterus Cervix (not routinely recommended)	Ovaries Adnexa Cervix (not routinely recommended)	Not mentioned

NT: nuchal translucency; LMP: last menstrual period; CRL: crown–rump length; IVF: in vitro fertilization, ART: assisted reproductive technology; BPD: biparietal diameter; HC: head circumference; AC: abdominal circumference; FL: femoral length, APAD: anterior–posterior abdominal diameter; TAD: transverse abdominal diameter; GIT: gastrointestinal tract, U/S: ultrasound.

**Table 2:** Combined first-trimester screening and specific examinations.

	AIUM	ASUM	AWMF	ISUOG	SOGC	WAPM
**Biochemistry**	Not mentioned	Maternal free β-hCG PAPP-A	Maternal free β-hCG PAPP-A	Maternal free β-hCG PAPP-A Recently, PlGF	Routinely combined with NT	Not mentioned
**NT**	Not mentioned	Upon request Trained staff According to FMF protocol CRL 45–84 mm	According to FMF protocol CRL 45–84 mm	Trained staff According to FMF protocol CRL 45–84 mm	Trained staff According to FMF protocol CRL 45–84 mm	Trained staff CRL 45–84 mm
**Additional ultrasound markers**	Not mentioned	Not mentioned	→ Tricuspid flow. → Ductus venosus flow. In intermediate-risk women: → Nasal bone.	→ Tricuspid flow. → Ductus venosus flow. → Nasal bone.	In high-risk pregnancies: → Tricuspid flow. → Ductus venosus flow. → Nasal bone not routinely recommended.	→ Tricuspid flow. → Cranial posterior fossa. → Ductus venosus flow (not routinely).
**Preeclampsia**	Not mentioned	Not mentioned	According to FMF protocol: → Maternal history. → Uterine artery PI. → Maternal biochemistry. → Biophysical examinations. If risk of >1:100, offer aspirin 150 mg daily (until 36 wks).	According to FMF protocol: → Maternal characteristics. → Medical history. → UtA-PI. → Serum PIGF. → MAP. For limited resources: → Maternal history. → Maternal characteristics. → MAP. If risk of >1:100, offer aspirin 150 mg daily (until 36 wks).	According to FMF protocol: → Maternal characteristics. → Medical history. → UtA-PI (not routinely). → MAP. → Maternal biochemistry (PAPP-A, PIGF).	Not mentioned
**FGR**	Not mentioned	Not mentioned	Doppler examinations	Not mentioned	→ Maternal characteristics. → Maternal history. → UtA-PI (not routinely). → MPA.	Not mentioned
**cfDNA**	Not mentioned	Not mentioned	In intermediate-risk women. After first-trimester screening. For inconclusive test: → Repeat in 2 weeks. Screening for rare, structural, microdeletions/duplications, or monogenic defects not recommended.	Screening for common aneuploidies. May be extended to screening for microdeletions, microduplications or other aneuploidies.	Not mentioned	Not mentioned
**Diagnostic procedures**	For fetal anomalies in early gestation	Not mentioned	Fetal malformations. NT ≥ 3.0 mm. Abnormal biochemistry. Abnormal cfDNA.	Not mentioned	For NT ≥ 3.5 mm	Not mentioned

β-hCG: β-human chorionic gonadotropin; PAPP-A: pregnancy-associated plasma protein A; PlGF: placental growth factor; NT: nuchal translucency; CRL: crown–rump length, FMF: Fetal Medicine Foundation; MAP: mean arterial pressure.

**Table 3:** Standard planes and images.

	AIUM	ISUOG	WAPM
**Head**	Axial/sagittal plane Cranial bones Falx cerebri Choroid plexus Thalami Posterior fossa Brain stem Intracranial translucency Cisterna magna	Axial/midsagittal view Calcification/shape of cranium Falx cerebri Choroid plexus (butterfly sign) Thalami Posterior fossa Brain stem Cerebral peduncles with aqueduct of Sulvius Intracranial translucency Cisterna magna	Axial/sagittal plane Cranial bones Falx cerebri Posterior fossa
**Face and profile**	Axial/sagittal/coronal/tangential plane Nasal bone Profile Maxilla Mandible	Midsagittal/axial/coronal plane Forehead Orbits Nasal bone Maxilla Retronasal triangle Upper lip Mandible	Axial/sagittal/coronal plane Profile Orbits Anterior palate
**Neck**	Axial/sagittal/coronal plane NT Abnormal fluid collections	Sagittal view NT Abdominal fluid collections	Sagittal view NT
**Heart and thorax**	Axial/sagittal/coronal plane Cardiac position, axis 4-chamber view with color 3-vessel trachea view with color Symmetric lungs Diaphragm demarcation	Axial view Cardiac position, axis and size 4-chamber view with color Left ventricular outflow tract view with color 3-vessel trachea view with color Color Doppler of ductus venosus Thoracic shape Symmetric lungs Diaphragmatic demarcation	Axial view Lungs fields Cardiac situs Size and position 4-chamber view 3-vessel view Arches
**Abdomen and pelvis**	Axial/sagittal/coronal plane Stomach Liver Cord insertion Bladder (color Doppler of umbilical arteries) Anterior wall Kidneys	Axial/sagittal view Stomach Bladder Abdominal wall Bladder (color Doppler of umbilical arteries) Kidneys	Axial/sagittal/coronal view Stomach Cord insertion Bladder (color Doppler of umbilical arteries) Kidneys Genital tubercle
**Limbs**	Presence of 4 extremities Presence of 3 long bones Presence of hands/feet	Presence of 4 extremities Presence of 3 long bones Free movement	Axial/sagittal plane Presence of 4 extremities Presence of 3 long bones Presence of hands/feet Free movement
**Spine**	Axial, longitudinal plane Vertebral alignment Skin edge	Sagittal plane Vertebral alignment Skin edge Shape and continuity	Sagittal/coronal plane Vertebral alignment Skin edge
**Placenta**	Axial/sagittal plane Position of placenta Umbilical cord insertion Texture	Position of placenta Umbilical cord insertion Size and texture	Not mentioned

NT: nuchal translucency.

## Data Availability

No new data were created or analyzed in this study. Data sharing is not applicable to this article.
